# Insulin signaling in the whole spectrum of GH deficiency

**DOI:** 10.20945/2359-3997000000188

**Published:** 2019-11-01

**Authors:** Heraldo Mendes Garmes, Alejandro Rosell Castillo

**Affiliations:** 1 Departamento de Clínica Médica Faculdade de Ciências Médicas Universidade Estadual de Campinas Campinas SP Brasil Divisão de Endocrinologia, Departamento de Clínica Médica, Faculdade de Ciências Médicas da Universidade Estadual de Campinas (FCM-Unicamp), Campinas, SP, Brasil

**Keywords:** GH deficiency, insulin sensitivity, euglicemic hyperinsulinemic clamp

## Abstract

GH is one of the insulin counterregulatory hormones which acts in the opposite way to insulin, increasing the glucose production by the liver and kidneys and decreasing glucose uptake from peripheral tissues, thus being a hyperglycemic hormone. When in excess, as in acromegaly, it induces glucose intolerance and diabetes. As expected, patients with GH deficiency (GHD) have hypoglycemia, especially in early childhood, but as GH is also a lipolytic hormone, these patients are becoming obese with higher percentages of body fat. Although obesity in general is directly related to insulin resistance, in patients with GH secretion disorders this relationship may be altered. In acromegaly there is a decrease in fat mass with worsening insulin sensitivity and mice with isolated GHD are characterized by greater insulin sensitivity despite excess fat mass. In humans with GHD, body composition shows increased body fat and decreased free fat mass, but the results regarding insulin sensitivity are still controversial in these patients. These discrepant results regarding insulin sensitivity in patients with GHD suggest the existence of other variables influencing these results. In the present review, we will try to follow the path of the different researches conducted on this subject, both in animal and human models, with the goal of understanding the current knowledge of insulin sensitivity across the spectrum of GHD. Arch Endocrinol Metab. 2019;63(6):582-91

## INTRODUCTION

GH deficiency (GHD) is characterized by signs and symptoms resulting from decreased serum GH levels that vary according to the age of patient. Adult GHD syndrome was first described in 1992 (1), as a consequence of diseases that compromise the anatomy and secretory function of pituitary somatotrophic cells or interfere with peripheral GH action. They have varied etiologies, from inherited genetic factors to acquired lesions, such as tumors, inflammatory processes and vascular lesions. The etiology or therapy of these varied etiologies can directly influence insulin sensitivity regardless of the decrease in serum GH levels. Adults with GHD may be classified according to stage of disease onset; when it begins in childhood, congenital diseases are the most common etiologies, being secondary to pituitary structural lesions in most cases who have onset in adulthood (2).

From a clinical standpoint in childhood GHD usually produces severe hypoglycemia, sometimes associated with seizures, prolonged hyperbilirubinemia and hypothermia. Height at birth is usually normal. After 12 months of age, there is usually short stature, with decreased growth velocity and delayed bone maturation (3). GHD in adults is not associated with a single symptom or pathognomonic sign, usually adults have nonspecific symptoms such as weakness, social isolation and difficulty concentrating. The most specific and striking changes are those of body composition with increased fat mass of 7% to 10% higher than expected for age, sex and height and decreased muscle mass, total body water and bone mineral density (4-6). GHD often overlaps with metabolic syndrome in relation to obesity and hyperlipidemia.

In this sense, we know that the percentage of fat tissue is increased in patients with GHD and that decreased insulin sensitivity (IS) is a characteristic of overweight and metabolic syndrome, but the relationship between IS and GHD is still controversial. In the context of GHD the results of animal studies have shown that there is a greater IS in this condition, while human studies have contradictory results. These discrepant results regarding IS in patients with GHD suggest the existence of other variables influencing these results. In this paper we review the complex physiology that influences glucose metabolism and IS in GHD patients, the results from animal and human studies about IS in GHD and discuss presumed factors that would justify the discrepancy of results in assessing IS in these patients

## INSULIN

Human insulin is a protein with a molecular weight of 5.808 Kda and is made up of two amino acid chains, linked via disulfide bonds (7). To exert its effects on target cells, insulin binds and activates a membrane protein receptor, which is formed by four subunits that are held together by disulfide bonds: two alpha subunits, which are located entirely on the outside of the cell membrane and two beta subunits, which penetrate through the membrane, projecting into the cell cytoplasm. Insulin couples to alpha subunits and induces phosphorylation of the intracellular portion of the receptor (7). Autophosphorylation of receptor beta subunits activates a local tyrosine kinase, which in turn causes phosphorylation of several other intracellular enzymes, including the group called insulin receptor substrates (8). The main effects of insulin stimulation are to promote glucose uptake in muscle and hepatic tissue and its storage in glycogen form, to further promote the conversion of excess glucose into fatty acids for lipogenesis, to inhibit hepatic glucose production and finally to promote protein synthesis and storage in muscle tissue (9).

Although several plasma factors play an important role in the control of insulin secretion, serum glucose concentration is the main regulator of its secretion, and increased blood glucose induces insulin release to restore normoglycemia. On the other hand, lowering glycemic levels rapidly induces interruption of insulin secretion and activates various counterregulation mechanisms in order to increase glucose levels. There is increased glucagon secretion by the pancreatic alpha cells, the hypothalamus stimulates the sympathetic nerve system and later both cortisol and GH are secreted in response to hypoglycemia. These hormones decrease glucose uptake by peripheral tissues and increase glucose production in the liver and kidneys, helping to prevent hypoglycemia (10,11).

## GROWTH HORMONE (GH)

The GH, also known as somatotropic hormone or somatotropin, is a protein that contains 191 single chain amino acids with a molecular weight of 22.005 Kda (12). It is produced by pituitary somatotrophs, and its secretion occurs in pulses that are controlled by the hypothalamus through GH-releasing hormone (GHRH), somatostatin and ghrelin (13,14). Somatostatin exerts an inhibitory effect, while GHRH and ghrelin stimulate GH secretion through different specific G protein-coupled receptors. GH pulses are greater at night and may not exist during the day, when their serum level may be undetectable, especially in obese and elderly people. Episodic release of GH increases with exercise and fasting. GH secretion is suppressed by increased glucose levels and is stimulated by insulin-induced hypoglycemia (15-20).

Upon secretion, GH binds to its peripheral receptors (GHRs) which belong to specifically class 1 cytokine receptor family. These receptors are expressed in various tissues of the organism especially the liver, cartilage, muscle, fat, pancreas and in the kidneys. Following binding of GH to the receptor, intracellular signal transduction is triggered by activation and phosphorylation of the enzyme janus kinase 2 (JAK2), resulting in the engagement of various intracellular signaling proteins, including signal transducers and activators of transcription (STAT), and mitogen-activated protein kinase (MAP) pathway components that in turn regulate target genes such as hepatic genes for the production of insulin-like growth factor-1 (IGF-1) (21-24). GH and IGF-1 are known to have independent actions in both growth and metabolism (25).

In addition to its general growth-provoking effect, growth hormone has several specific metabolic effects including increased protein synthesis; increased fatty acid mobilization of adipose tissue inducing increased blood level of fatty acids and increased use of fatty acids as a source of energy. There is also reduced utilization of glucose and increased hepatic and renal glucose production (26-30). These changes result in growth hormone-induced insulin resistance that favors increased blood glucose. The ability of GH to promote the catabolic effect on adipose tissues along with the anabolic effect on muscle tissue induces increased lean mass.

The IGF-1 produced in the liver by GH stimulation suppresses GH secretion by negative feedback and negativaly regulates GH receptors through paracrine action (31). IGF-I, also known as somatomedine C, belongs to the IGF system, which consists of different elements: IGF-I and IGF-2, two receptor types: IGFR-1 and IGFR-2 and various binding proteins IGFBP 1 to 6. The IGFs have a high degree of structural homology with insulin, which has an acceptable affinity for IGFR-1 and the IGF-1 also has affinity for insulin receptor (32). IGF-1 has an important effect on carbohydrate metabolism, and studies have shown that IGF-1 can exert insulin-like effects on blood transport and glucose concentrations (33-35). It is noteworthy that it is not yet well understood which effects on carbohydrate metabolism depend on the action of GH or IGF-1.

## INSULIN SENSITIVITY

The concept of IS was introduced by Sir Harold Himsworth in 1939 when studying the response of diabetic patients to glycemic and insulin stimulus (9). Insulin resistance is defined as a decrease in the sensitivity of peripheral tissues (skeletal muscle, adipose and liver) to insulin actions, it is a metabolic state in which target cells have insufficient response to normal levels of circulating insulin, thus being an important predictor of Diabetes Mellitus type 2 development (9-11).

## INSULIN SENSITIVITY ASSESSMENT METHOD

To assess IS many methods have been developed, it can be accessed through direct methods or indirect markers. Direct evaluation can investigate the action of exogenous insulin, as in the euglycemic hyperinsulinemic clamp (EHC) or endogenous insulin released from a stimulus as in the glucose tolerance test (TTG) and Bergman’s minimal model, hyperglycemic clamp, oral glucose tolerance test (OGTT) or with foods such as mixed meal tolerance test (MTT) (36). The EHC is considered the gold standard to investigate IS (37), since the other tests are influenced by several factors involved in the glucose metabolism such as pancreatic alpha and beta cell function, incretinic and counterregulators hormones and hepatic glucose production (36,38,39). However, the cost, technical difficulties and execution time make it difficult to use the EHC in clinical practice and even in clinical research. Thus, several authors seek indirect markers that are more accessible for investigating insulin sensitivity as homeostatic model assessment insulin resistance (HOMA-IR) (38).

Patients with multiple pituitary deficiency have impaired glucose metabolism and cannot increase the production of counter-regulating hormones, such as cortisol and GH, when blood glucose decreases. Because of this, they cannot increase glucose production in the context of hypoglycemia. Therefore, to assess IS in patients with GHD, a method that removes the influence of counterregulatory hormones on glucose production and uptake by the liver and peripheral tissues should be used. As previously explained, EHC is a good method for investigating IS which, due to its hyperinsulinemic state induces suppression of endogenous glucose production, removing the influence of the absence of counterregulatory hormones and does not depend on an insulin secretion which can be impaired in GHD patients (40). It only evaluates insulin-dependent glucose uptake, faithfully representing the sensitivity to insulin action in these patients. In this regard, studies in humans have already demonstrated the lack of correlation between EHC and other methods used to assess IS in patients with GHD (41,42) suggesting that using other methods may induce controversial results. Therefore, in this review we considered only scientific studies that used HEC as a method for assessing IS in patients with GHD.

BMI is known to be inversely related to IS in humans and GHD patients have increased fat mass and decreased free fat mass. In this sense, it is important to compare IS data in patients with GHD with a control group paired by BMI, as well as age and gender that also influence the assessment of IS. Therefore, we only use in this review studies that evaluated IS in GHD patients and compared them to age, sex and BMI matched groups, avoiding controversial results.

## INSULIN SENSITIVITY IN ANIMAL MODELS

In the 1990s in order to better understand the physiological effects of growth hormone, a strain of GHR receptor (GHR -/-) disrupted mice was generated in the laboratory. These mice are insensitive to GH and have low levels of IGF-1 and high GH. They are small and the organs are also proportionally small. In addition to these characteristics, GHR -/- mice have low insulin and glucose levels and are sensitive to insulin, although they are glucose intolerant due to pancreatic islet size reduction. One of the most interesting features is that these mice have a longer life expectancy and are resistant to various cancers (40,43-45). More recently other mouse strains have been developed: an onset isolated GHD (AOiGHD) model with selective acquired destruction of somatotrophic cells in the anterior pituitary and a model that presents a genetic mutation in the gene encoding GH (GH -/-), with both presenting low GH and IGF-1 levels (46,47). These mouse strains, like the GHR -/- strain have low levels of insulin and glycemia, are extremely sensitive to insulin when compared to controls despite having a higher amount of fat, and are also intolerant to glucose due to a decrease in size of the pancreatic islets (46,47). It is noteworthy that in these studies the method used for IS evaluation was the insulin tolerance test (ITT) (40,43-47). On the other hand, other studies in transgenic animals with global antagonism in GH (GHa) and AOiGHD using EHC for IS evaluation showed similar IS in relation to controls (48-50).

All of these animal models have been very helpful in better understanding the effects and functions of GH aiming to establish a similarity with what happens in humans. GHR -/- animal models have the pattern of Laron Syndrome, AOiGHD would be equivalent to acquired isolated GHD in adults and the newer GH -/- animal model would simulate what happens in isolated GHD syndrome. Finally, the authors of these studies observed that there is a consensus in animal model studies that non-action of GH would lead to decreased insulin and glucose levels, glucose intolerance and increased IS despite having a higher percentage of fat (47,51) (Table 1).

**Table 1:** Insulin sensitivity (IS) in some types of animals without GH signaling

Autor	Mouse line	Tissue Disrupted	Matched Controls	GH	IGF-1	Free Fat Mass	Fat Mass	Glicose	Insulin	IS
Coschigano KT, 2003 (43)	GHR-/-	Global	Yes	↑	↓	↓	↑	↓	↓	↑
Liu JL, 2004 (40)	GHR-/-	Global	Yes	↑	↓	↓	↑	↓	↓	↑
Luque RM, 2011 (46)	AOiGHD	Somatotroph cells	Yes	↓	↓	↓	↑	↓	↓	↑
Lubbers ER, 2013 (44)	GHR-/-	Global	Yes	↑	↓	↓	↑	↓	↓	↑
Junnila RK, 2016 (45)	GHR-/-	Global	Yes	↓	↓	↓	↑	↓	↓	↑
List EO, 2019 (47)	GH-/-	Global	Yes	↓	↓	↓	↑	↓	↑	↑
Haluzik M, 2003 (48)	GHa	Global	Yes	↑	↓	↓	↑	↓	↓	=
Yakar S, 2004 (49)	GHa	Global	Yes	↑	↓	↓	↑	↓	↓	=
Cordoba-Chacon J, 2014 (50)	AOiGHD	Somatotroph cells	Yes	↓	↓	↓	↑	↓	↓	=

GHR-/-: animals with mutation in the gene encoding GH receptor. AOiGHD: adult onset isolated GH deficiency animals. GH -/-: animals with mutation in the gene encoding GH. GHa: animals with global antagonism in GH.

## INSULIN SENSITIVITY IN GHD FROM CONGENITAL DISEASES

In a human cohort with a mutation of the GHRH receptor gene, known as isolated GHD (IGHD), GH and IGF-1 levels are significantly decreased from birth. Although these patients have normal size at birth, they evolve with low growth velocity and severe short stature. IGHD patients have a significant increase in visceral fat (52), but IS is increased in relation to age, sex and BMI matched control group when evaluated by HOMA-IR and similar to control group when assessed by glucose and insulin curves from GTT, clearly showing that patients with IGHD do not have insulin resistance (53). It is noteworthy that, in this same study, the authors also showed that these patients have decreased HOMA-β, indicating lower insulin secretion capacity, justifying a higher percentage of patients with glucose intolerance when compared to the control group.

Regarding patients with GH resistance due to multiple inactivating mutation on the GH receptor gene, Laron Syndrome, some regions of the world concentrate a larger number of patients. In the Israeli cohort, body composition showed excess percentage of body fat associated with low muscle and bone mass (54). Patients had frequent hypoglycemia in early childhood and blood glucose levels were lower than in the normal population, with higher serum insulin levels for concomitant glucose level (55). In the Ecuadorian cohort, despite obesity, patients had lower blood glucose, insulin and HOMA-IR values, indicating better insulin sensitivity and had a lower incidence of diabetes than their relatives (56). Interestingly, a study comparing these patients with a group of patients with intrauterine growth retardation, severe short stature, decreased pancreatic reserve, but normal GH signaling demonstrated its importance for the presence of insulin resistance, since this group of patients with normal signaling presented insulin resistance and early onset type 2 diabetes (57).

## INSULIN SENSITIVITY IN ACROMEGALY AND AFTER GH REPLACEMENT

The classic counterregulatory effect of insulin exerted by GH justifies the high rates of glucose intolerance and diabetes mellitus found in patients with acromegaly, where excess GH induces insulin resistance (58-61). GH has a lipolytic effect, increasing serum FFA levels that could compromise insulin action. The patient with acromegaly is an example of a patient with low fat mass percentage and insulin resistance. Excess circulating FFA and insulin resistance would induce beta cell failure over time, favoring glucose intolerance present in more than 50% of patients with new diagnosis of acromegaly (62).

In turn, GH replacement treatment in patients with GHD also seems to induce decreased IS. Although some studies have shown increased IS with GH replacement (63), most studies using HEC as an evaluation method have shown worsening IS with GH therapy in both adults (64-66) and children (67,68) evidencing the role of GH as a counterregulatory hormone. Also regarding treatment, it is noteworthy that in adults with GHD, low doses of GH appear to exert less insulin counterregulatory effect than classic doses aiming to normalize IGF-1 (69), and these contrarregulatory effects are more evident in obese and older (70).

Regarding the mechanisms involved in GH-induced SI worsening, a 2 x 2 factorial design study using the EHC method showed that acipimox, a lipolysis-blocking drug, prevents GH-induced IS worsening in patients with GHD (71). These results have been confirmed by other authors, suggesting that the decrease in IS caused by GH may be related to its lipolytic effect that increases serum levels of free fatty acid and intramyocellular triglyceride content, worsening IS (72-73). In this context it is plausible that obese GHD patients have low FFA turnover as an explanation for normal insulin sensitivity. The mechanisms involved in the relationship between FFA and insulin resistance were initially associated with competition between FFA and pyruvate substrates, inhibiting the glycolytic pathway (74). This evidence was later confirmed in a study that evaluated the effects of GH on IS using the EHC method (75). In addition, it is believed that FFA levels act on the insulin signaling pathway, inhibiting of insulin receptor substrate (IRS-1) and PI3K in the skeletal muscle and liver, which results in reduced GLUT4 translocation, leading to decreased IS (76,77).

Specifically in skeletal muscle, GH promotes the uptake of FFA through increased activity of lipoprotein lipase (78) and accumulate intramyocellular triglyceride content (73). In this process there is an accumulation of diacylglycerol and ceramides in skeletal muscle. It is known that these lipid intermediates inhibit insulin signaling pathways. In this sense, studies showed that diacylglycerol inhibits IRS-1 through the activation of the protein kinase C theta that induces insulin receptor phosphorylation in serine leading to insulin resistance. Ceramide inhibits an important mediator of insulin signaling pathway such as Akt/protein kinase B, an important mediator of insulin signaling pathway (79).

In adipose tissue, GH suppresses glucose uptake, as it has been shown that expression of the GLUT1 and GLUT4 transporters in the adipocyte cell membrane has been suppressed after GH administration (80). This mechanism is regulated by a subunit called p85, which negatively regulates PI3K-dependent insulin signaling, which in turn is important for the transfer and expression of GLUT1 and GLUT4 transporters, inducing insulin resistance (81).

In hepatocytes, the increase in GH-induced FFA uptake leads to an increase in lipid oxidation and accumulation of acetyl coenzyme A. This coenzyme induces an increase in blood glucose levels through the stimulation of two enzymes that participate in glyconeogenesis such as pyruvate carboxylase and phosphenolpyruvate carboxykinase and it stimulate the glucose 6 phosphatase, which increases the release of glucose in the liver (82).

Regarding the hyperglycemic effect, some studies do not show increased incidence of diabetes in patients using GH, but pharmacoepidemiological studies with a large number of patients showed a small increase in incidence of type 2 diabetes mellitus when compared to the normal population, especially in children with risk factors for diabetes. such as family history, corticoid use and obesity (83-85).

## INSULIN SENSITIVITY IN GHD

As shown in Table 2, the literature is controversial regarding the results of IS in patients with GHD. Some studies show that these patients have insulin resistance (86-90) and others show that IS is similar to the gender, age and BMI matched controls (41,42,88,90-92).

**Table 2:** Insulin sensitivity (IS) by euglicemic hyperinsulinemic clamp in GHD patients

Study	Number of cases	Number-of females	Number-of controls	Age	Controls matched	Glucose	Insulin	IS
Johansson JO, 1995 (86)	15	4	15	20-62	Yes	=	=	↓
Hew FL, 1996 (87)	14	4	12	43.2 ± 3.2	Yes	=	=	↓
Pincelli AI, 2001(90)	10	6	6	31-49	No	↓	=	=
Bülow B, 2004 (91)	11	10	11	25-33	Yes	=	↑	=
Ukropec J, 2008 (88)	9	3	9	28,1 ± 1,9	Yes	=	=	↓
Ukropec J, 2008 (88)	7	3	7	31,2 ± 3,2	Yes	=	=	=
Krusenstjerna-HT, 2011 (92)	8	0	8	42-62	Yes	=	↑	=
Balaz M, 2014 (89)	17	6	17	28-34	Yes	=	=	↓
Ciresi A, 2018 (41)	23	8	12	7-10	Yes	=	=	=
Castillo AR, 2019 (42)	15	6	15	22-56	Yes	↓	↓	=

These discrepancies in study results may be related to several factors, such as the etiologies of hypopituitarism that are related to decreased IS regardless of decreased GH levels, as in patients with craniopharyngioma who have more incidence of metabolic syndrome (93). Another factor that could influence IS would be hormone replacement therapy, especially corticosteroid replacement, but also thyroid and sexual hormone replacement. It is known that corticosteroid therapy may induce elevations in circulating glucose and insulin levels (94) and that the dose of corticosteroids used in these patients has decreased in current guidelines compared to older ones. In this sense, the two studies that used EHC to assess IS, published in the 1990s (86,87), used an average cortisone dose of 25 mg/day slightly above the recommended dose by current guidelines (95).

Obesity could also influence IS assessments in GHD patients, since the study using EHC has demonstrated that IS is similar to the control group matched for BMI, age and gender when patients are classified as obese and is decreased when the thin patients are compared to their paired controls (88).

Decreased lean mass and physical performance are associated with GHD, however, despite the known beneficial effects of physical activity on insulin sensitivity, there are few studies relating the effects of exercise on IS in GHD patients. In this sense, a study in patients with functional GHD after bariatric surgery showed that a physical activity program prevented deterioration in glucose metabolism after GH use for 6 months (96).

Another important factor that may lead to controversial results is the method used to assess IS. This review has already discussed the reasons why EHC is the most reliable method for assessing IS in patients with disorders in the production of counterregulatory hormones such as hypopituitarism. As an example we can cite studies from our group, we showed increased insulin sensitivity in patients with GHD compared to control group paired by age, gender and BMI using HOMA-IR (97), but the IS was similar in both groups using EHC (42). However, performing EHC and evaluating its results requires special care, it is noteworthy the importance of similar serum levels of hyperinsulinemia during clamp between the patient group and the control, making the blockade of tissue glucose production similar in both groups avoiding possible interference in the results from the action of counterregulating hormones, which is present only in the control group. Thus the evaluation of insulin sensitivity is performed under equal conditions in both groups.

Another factor, little discussed in the literature, that could influence these discrepancies could be the type of fat tissue present in patients with GHD. The relationship between fat tissue and IS is different between patients with normal GH levels and those with GH deficiency. While obesity is directly related to insulin resistance in healthy humans, this relationship is compromised in patients with GH deficiency. Despite the classic increase in fat mass in patients with GHD, the lack of decreased IS in these patients could be justified by the need for a minimum circulating GH level to act in the fat tissue to promote insulin resistance. In addition, animal studies have shown that the transfer of visceral fat tissue from GH receptor mutated mice to normal mice induces improved insulin sensitivity in these animals (98). Interestingly, evaluation of IS by the EHC method in patients with GHD showed that patients with onset of GHD in early life had better IS when compared with controls paired by sex, age, and BMI, while patients with hypopituitarism who started hormone deficiency later in life have IS similar to the control group, suggesting that adipose tissue that did not undergo the physiological action of GH during early childhood should be different from adipose tissue that experienced this action at this stage of life, since the former is more sensitive to insulin action, results not yet published (99).

In this regard, animal studies showed that the secretion of adipokines from fat tissue is different between GH receptor mutated rats and normal rats (100). The removal of visceral fat in these mutated rats induces worsening IS, whereas in normal rats it induces improvement, showing opposite differences in the characteristics of these fatty tissues in modulating IS (100).

It is noteworthy that EHC is a complex and expensive method, so in most studies the number of patients is small and because of this, minor variations in the population studied could influence the evaluation of IS, changing the results and favoring conflicting conclusions.

## CONCLUSION

In this review, we describe the physiological basis for understanding insulin signaling in the whole spectrum of GH deficiency, showing the conflicting results of scientific studies in this regard, and discuss factors that could influence these results. Finally, we point out that there is no evidence in the literature to classify GHD patients in general as having insulin resistance, despite the well-established increase in fat mass present in these patients. Further studies are needed for a better understanding of IS in patients with GHD and the factors that may influence the results of this evaluation, such as those related to the characteristics of patients with GHD and to the methodology for IS evaluation.
